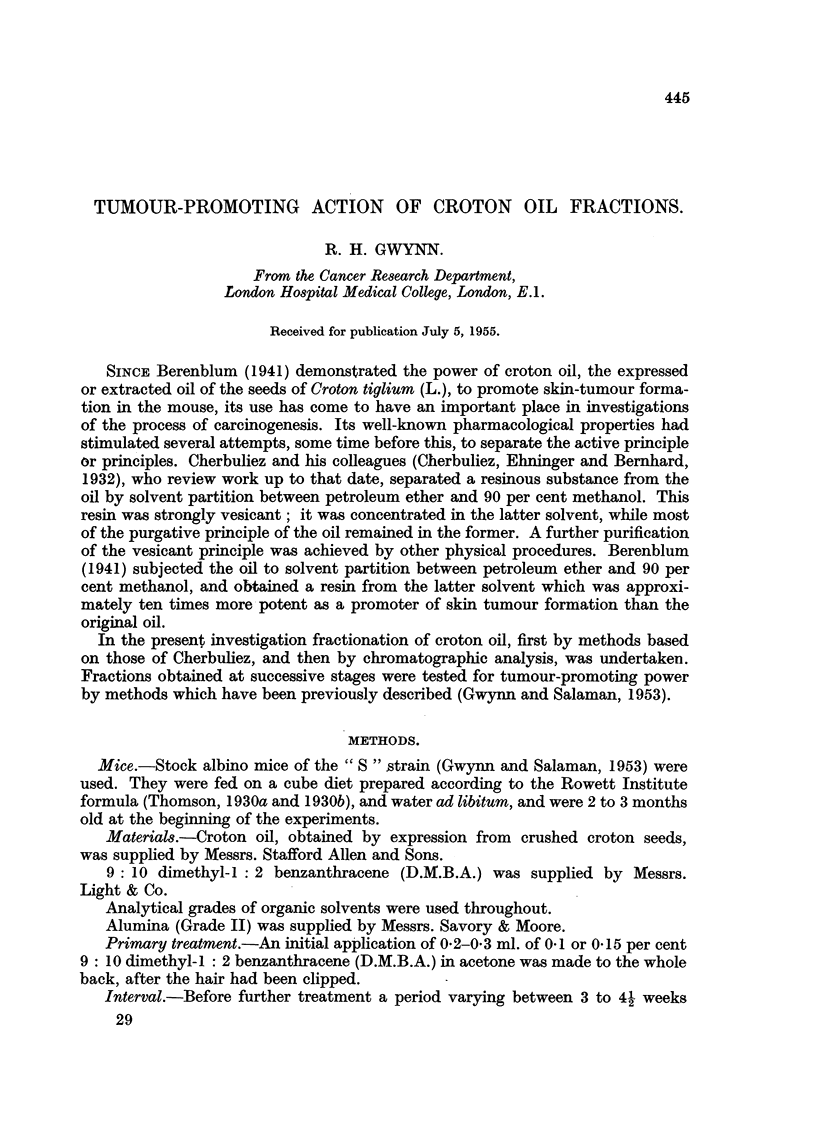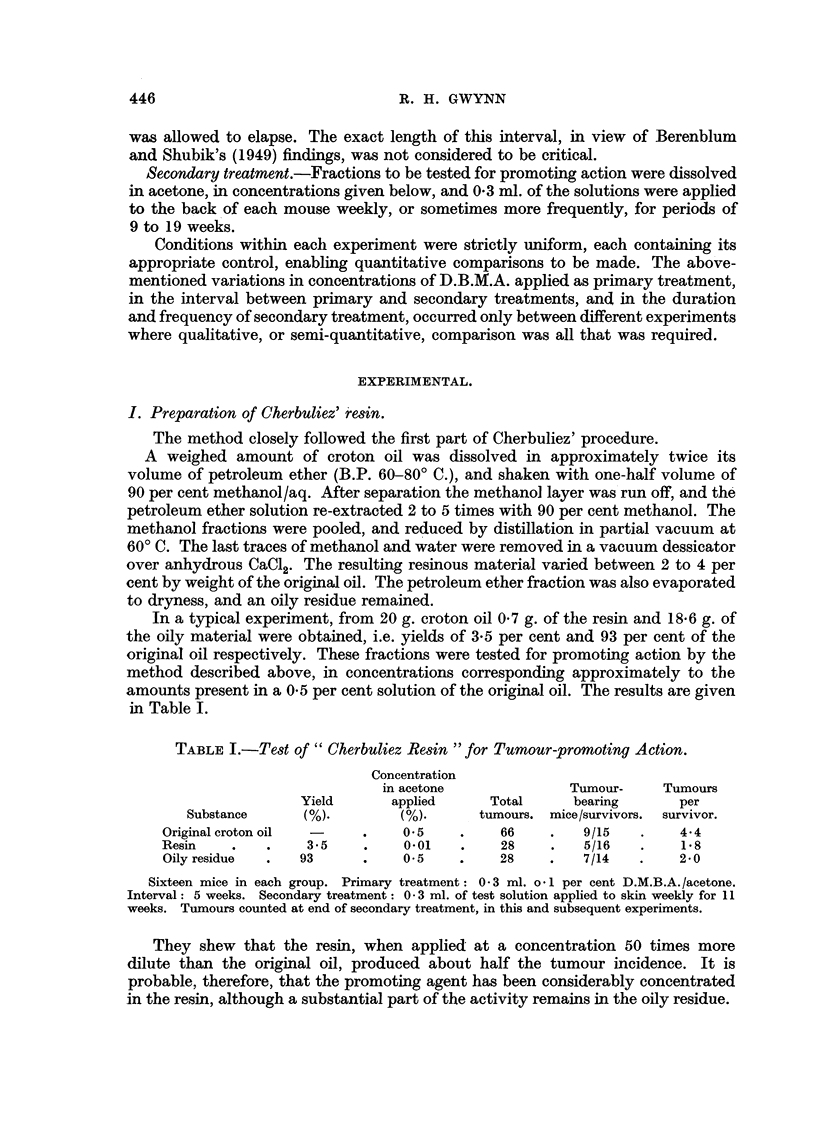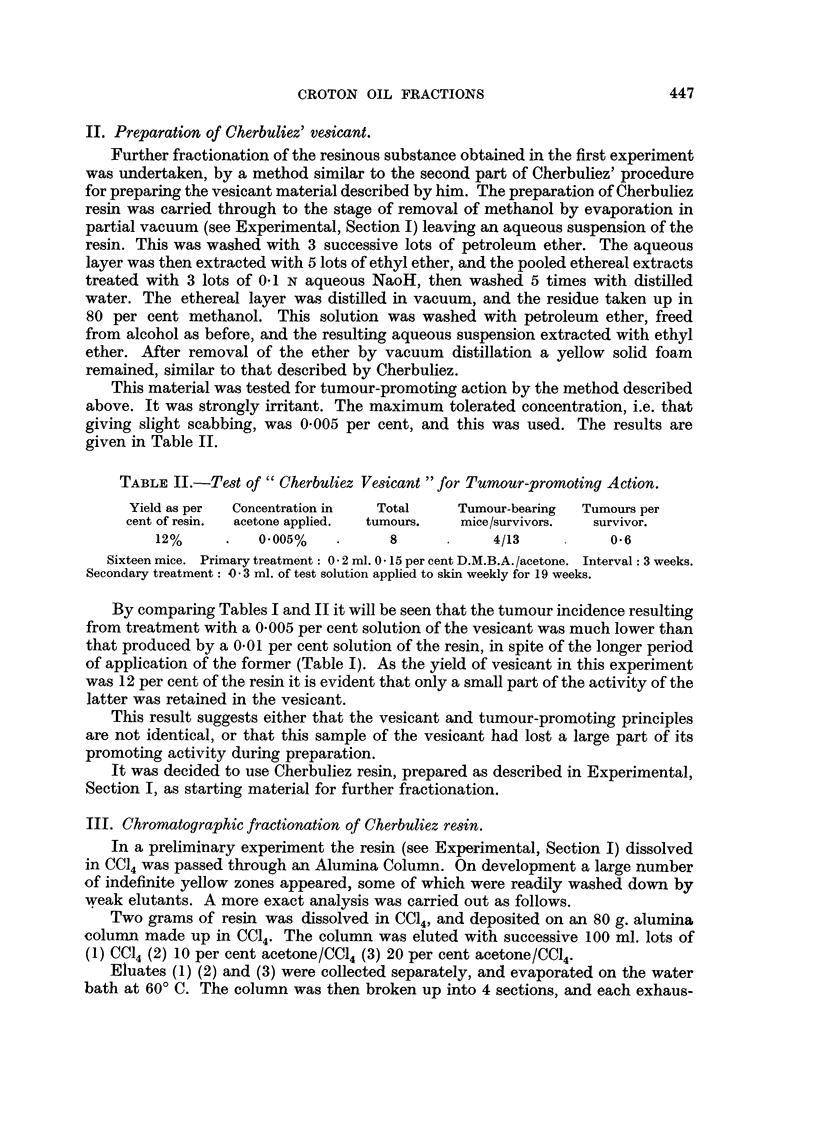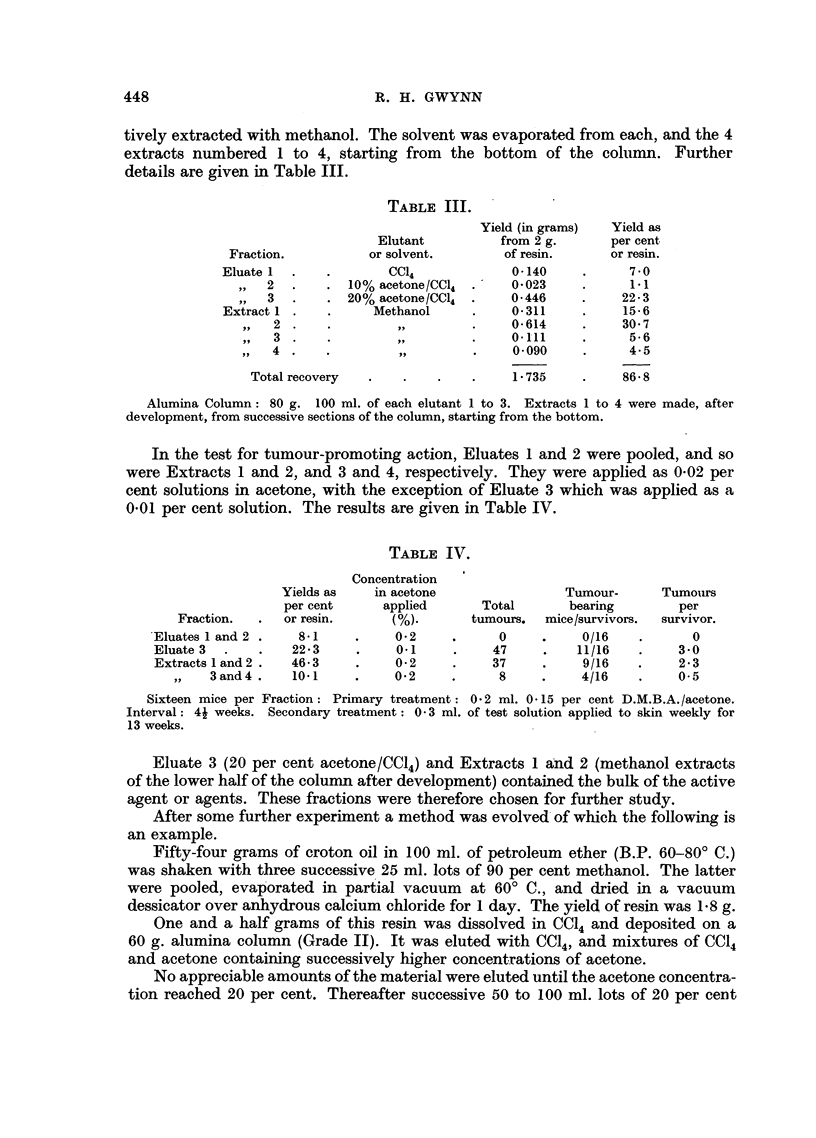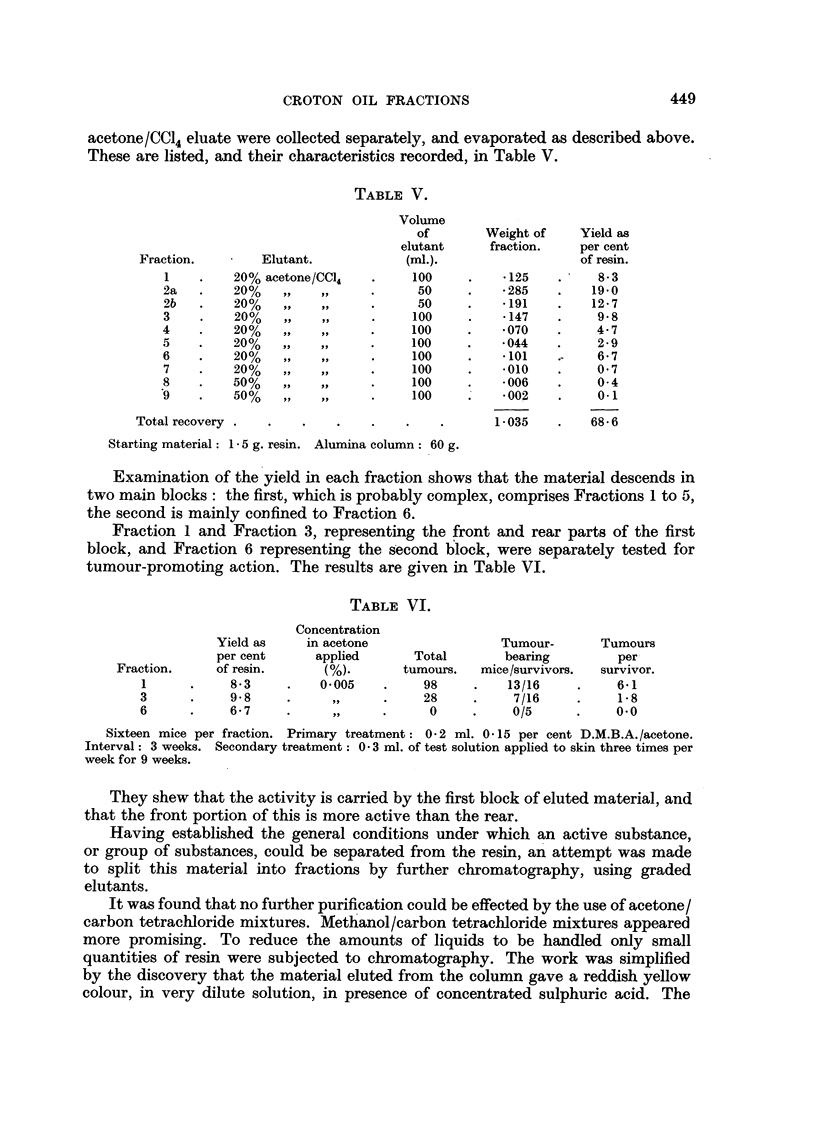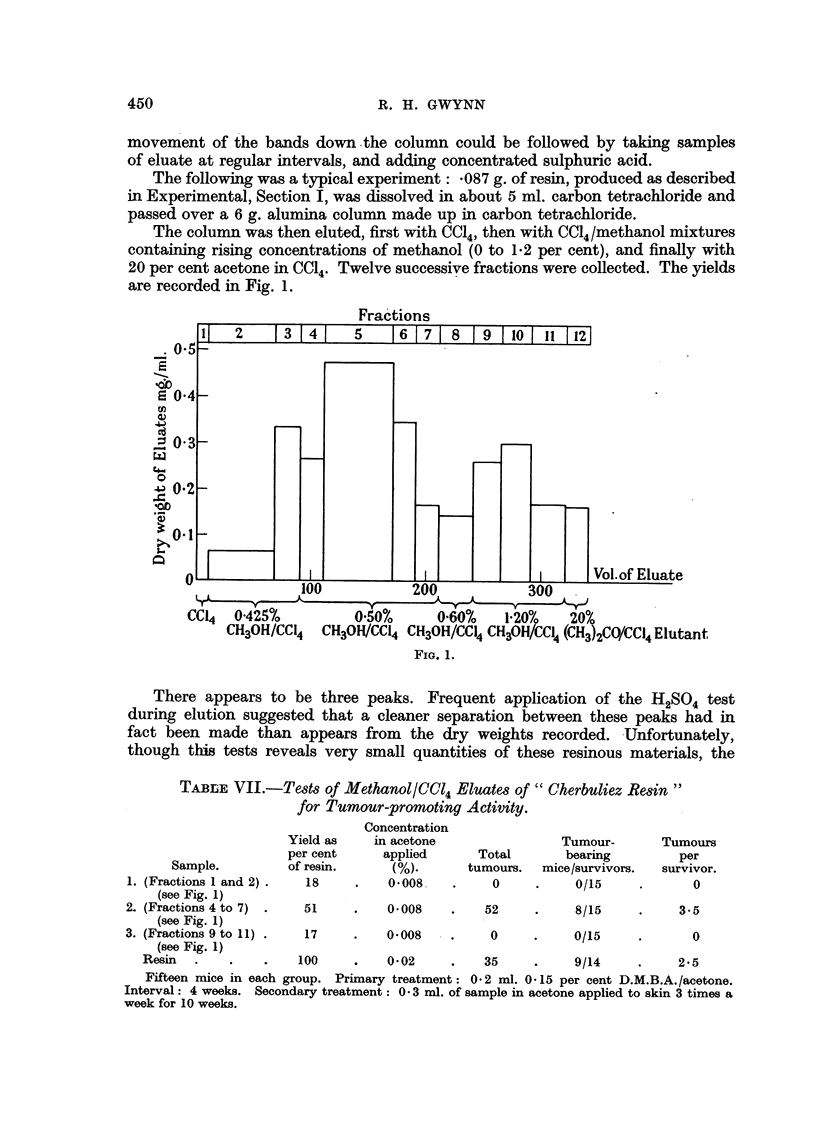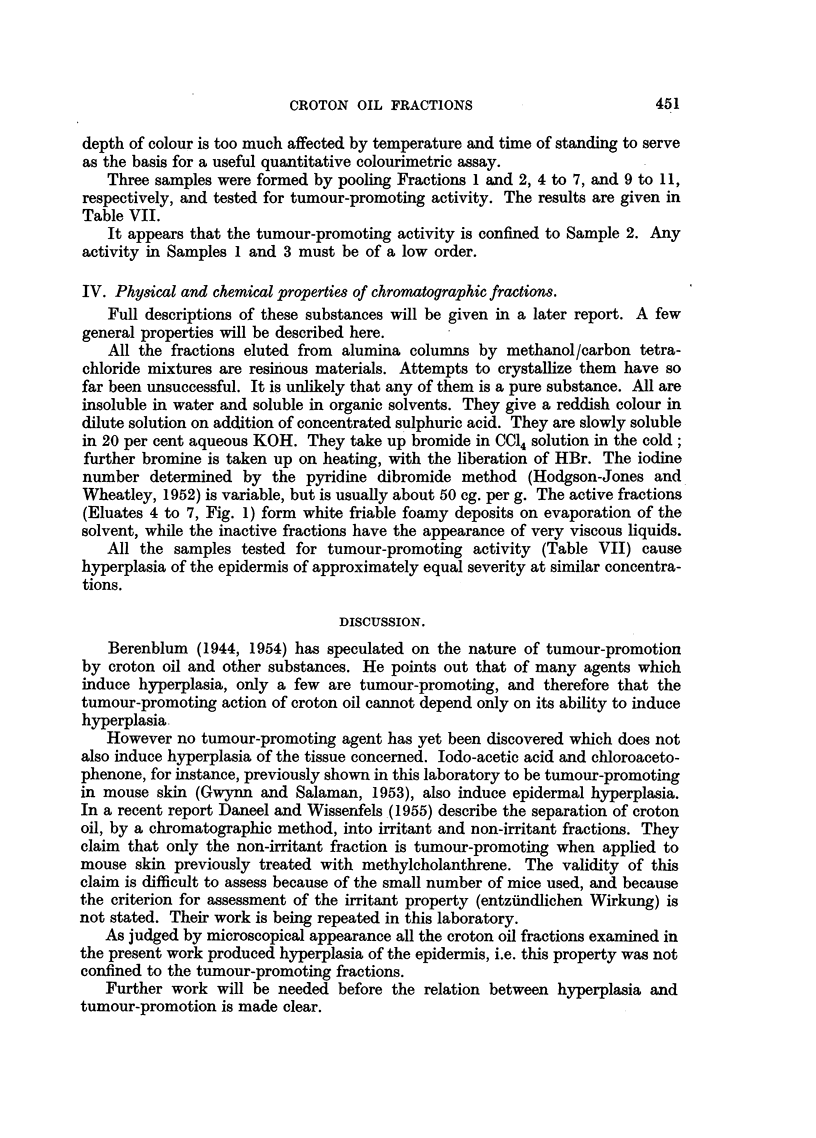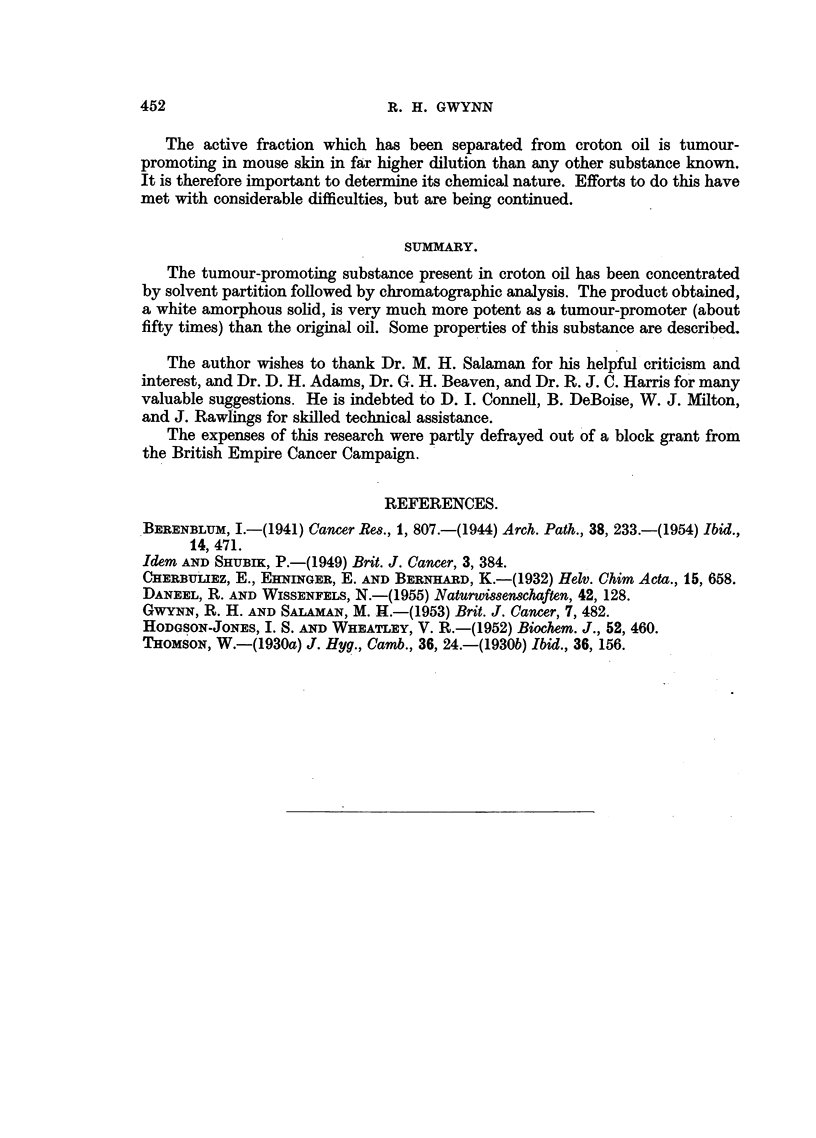# Tumour-promoting Action of Croton Oil Fractions

**DOI:** 10.1038/bjc.1955.44

**Published:** 1955-09

**Authors:** R. H. Gwynn


### TUMOUR-PROMOTING ACTION OF CROTON OIL FRACTIONS.

R. H. GWYNN.

*From the Cancer Research Department,*
*London Hospital Medical College, London, E.1.*

Received for publication July 5, 1955.

SINCE Berenblum (1941) demonstrated the power of croton oil, the expressed or extracted oil of the seeds of *Croton tiglium* (L.), to promote skin-tumour formation in the mouse, its use has come to have an important place in investigations of the process of carcinogenesis. Its well-known pharmacological properties had stimulated several attempts, some time before this, to separate the active principle or principles. Cherbuliez and his colleagues (Cherbuliez, Ehninger and Bernhard, 1932), who review work up to that date, separated a resinous substance from the oil by solvent partition between petroleum ether and 90 per cent methanol. This resin was strongly vesicant; it was concentrated in the latter solvent, while most of the purgative principle of the oil remained in the former. A further purification of the vesicant principle was achieved by other physical procedures. Berenblum (1941) subjected the oil to solvent partition between petroleum ether and 90 per cent methanol, and obtained a resin from the latter solvent which was approximately ten times more potent as a promoter of skin tumour formation than the original oil.

In the present investigation fractionation of croton oil, first by methods based on those of Cherbuliez, and then by chromatographic analysis, was undertaken. Fractions obtained at successive stages were tested for tumour-promoting power by methods which have been previously described (Gwynn and Salaman, 1953).

### METHODS.

*Mice.*—Stock albino mice of the "S" strain (Gwynn and Salaman, 1953) were used. They were fed on a cube diet prepared according to the Rowett Institute formula (Thomson, 1930*a* and 1930*b*), and water *ad libitum*, and were 2 to 3 months old at the beginning of the experiments.

*Materials.*—Croton oil, obtained by expression from crushed croton seeds, was supplied by Messrs. Stafford Allen and Sons.

9 : 10 dimethyl-1 : 2 benzanthracene (D.M.B.A.) was supplied by Messrs. Light & Co.

Analytical grades of organic solvents were used throughout.

Alumina (Grade II) was supplied by Messrs. Savory & Moore.

*Primary treatment.*—An initial application of 0·2–0·3 ml. of 0·1 or 0·15 per cent 9 : 10 dimethyl-1 : 2 benzanthracene (D.M.B.A.) in acetone was made to the whole back, after the hair had been clipped.

*Interval.*—Before further treatment a period varying between 3 to 4½ weeks was allowed to elapse. The exact length of this interval, in view of Berenblum and Shubik's (1949) findings, was not considered to be critical.

*Secondary treatment.*—Fractions to be tested for promoting action were dissolved in acetone, in concentrations given below, and 0·3 ml. of the solutions were applied to the back of each mouse weekly, or sometimes more frequently, for periods of 9 to 19 weeks.

Conditions within each experiment were strictly uniform, each containing its appropriate control, enabling quantitative comparisons to be made. The above-mentioned variations in concentrations of D.B.M.A. applied as primary treatment, in the interval between primary and secondary treatments, and in the duration and frequency of secondary treatment, occurred only between different experiments where qualitative, or semi-quantitative, comparison was all that was required.

### EXPERIMENTAL.

### *I. Preparation of Cherbuliez' resin.*

The method closely followed the first part of Cherbuliez' procedure.

A weighed amount of croton oil was dissolved in approximately twice its volume of petroleum ether (B.P. 60–80° C.), and shaken with one-half volume of 90 per cent methanol/aq. After separation the methanol layer was run off, and the petroleum ether solution re-extracted 2 to 5 times with 90 per cent methanol. The methanol fractions were pooled, and reduced by distillation in partial vacuum at 60° C. The last traces of methanol and water were removed in a vacuum dessicator over anhydrous $CaCl_2$. The resulting resinous material varied between 2 to 4 per cent by weight of the original oil. The petroleum ether fraction was also evaporated to dryness, and an oily residue remained.

In a typical experiment, from 20 g. croton oil 0·7 g. of the resin and 18·6 g. of the oily material were obtained, i.e. yields of 3·5 per cent and 93 per cent of the original oil respectively. These fractions were tested for promoting action by the method described above, in concentrations corresponding approximately to the amounts present in a 0·5 per cent solution of the original oil. The results are given in Table I.

TABLE I.—*Test of " Cherbuliez Resin " for Tumour-promoting Action.*

| Substance | Yield (%). | Concentration in acetone applied (%). | Total tumours. | Tumour-bearing mice/survivors. | Tumours per survivor. |
|---|---|---|---|---|---|
| Original croton oil | — | 0·5 | 66 | 9/15 | 4·4 |
| Resin | 3·5 | 0·01 | 28 | 5/16 | 1·8 |
| Oily residue | 93 | 0·5 | 28 | 7/14 | 2·0 |

Sixteen mice in each group. Primary treatment: 0·3 ml. 0·1 per cent D.M.B.A./acetone. Interval: 5 weeks. Secondary treatment: 0·3 ml. of test solution applied to skin weekly for 11 weeks. Tumours counted at end of secondary treatment, in this and subsequent experiments.

They shew that the resin, when applied at a concentration 50 times more dilute than the original oil, produced about half the tumour incidence. It is probable, therefore, that the promoting agent has been considerably concentrated in the resin, although a substantial part of the activity remains in the oily residue.

II. *Preparation of Cherbuliez' vesicant.*

Further fractionation of the resinous substance obtained in the first experiment was undertaken, by a method similar to the second part of Cherbuliez' procedure for preparing the vesicant material described by him. The preparation of Cherbuliez resin was carried through to the stage of removal of methanol by evaporation in partial vacuum (see Experimental, Section I) leaving an aqueous suspension of the resin. This was washed with 3 successive lots of petroleum ether. The aqueous layer was then extracted with 5 lots of ethyl ether, and the pooled ethereal extracts treated with 3 lots of 0·1 N aqueous NaoH, then washed 5 times with distilled water. The ethereal layer was distilled in vacuum, and the residue taken up in 80 per cent methanol. This solution was washed with petroleum ether, freed from alcohol as before, and the resulting aqueous suspension extracted with ethyl ether. After removal of the ether by vacuum distillation a yellow solid foam remained, similar to that described by Cherbuliez.

This material was tested for tumour-promoting action by the method described above. It was strongly irritant. The maximum tolerated concentration, i.e. that giving slight scabbing, was 0·005 per cent, and this was used. The results are given in Table II.

TABLE II.—*Test of "Cherbuliez Vesicant" for Tumour-promoting Action.*

| Yield as per cent of resin. | Concentration in acetone applied. | Total tumours. | Tumour-bearing mice/survivors. | Tumours per survivor. |
|---|---|---|---|---|
| 12% | 0·005% | 8 | 4/13 | 0·6 |

Sixteen mice. Primary treatment : 0·2 ml. 0·15 per cent D.M.B.A./acetone. Interval : 3 weeks.
Secondary treatment : 0·3 ml. of test solution applied to skin weekly for 19 weeks.

By comparing Tables I and II it will be seen that the tumour incidence resulting from treatment with a 0·005 per cent solution of the vesicant was much lower than that produced by a 0·01 per cent solution of the resin, in spite of the longer period of application of the former (Table I). As the yield of vesicant in this experiment was 12 per cent of the resin it is evident that only a small part of the activity of the latter was retained in the vesicant.

This result suggests either that the vesicant and tumour-promoting principles are not identical, or that this sample of the vesicant had lost a large part of its promoting activity during preparation.

It was decided to use Cherbuliez resin, prepared as described in Experimental, Section I, as starting material for further fractionation.

III. *Chromatographic fractionation of Cherbuliez resin.*

In a preliminary experiment the resin (see Experimental, Section I) dissolved in $CCl_4$ was passed through an Alumina Column. On development a large number of indefinite yellow zones appeared, some of which were readily washed down by weak elutants. A more exact analysis was carried out as follows.

Two grams of resin was dissolved in $CCl_4$, and deposited on an 80 g. alumina column made up in $CCl_4$. The column was eluted with successive 100 ml. lots of (1) $CCl_4$ (2) 10 per cent acetone/$CCl_4$ (3) 20 per cent acetone/$CCl_4$.

Eluates (1) (2) and (3) were collected separately, and evaporated on the water bath at 60° C. The column was then broken up into 4 sections, and each exhaustively extracted with methanol. The solvent was evaporated from each, and the 4 extracts numbered 1 to 4, starting from the bottom of the column. Further details are given in Table III.

TABLE III.

| Fraction. | Elutant or solvent. | Yield (in grams) from 2 g. of resin. | Yield as per cent or resin. |
|---|---|---|---|
| Eluate 1 | $CCl_4$ | 0·140 | 7·0 |
| ,, 2 | 10% acetone/$CCl_4$ | 0·023 | 1·1 |
| ,, 3 | 20% acetone/$CCl_4$ | 0·446 | 22·3 |
| Extract 1 | Methanol | 0·311 | 15·6 |
| ,, 2 | ,, | 0·614 | 30·7 |
| ,, 3 | ,, | 0·111 | 5·6 |
| ,, 4 | ,, | 0·090 | 4·5 |
| Total recovery | | 1·735 | 86·8 |

Alumina Column: 80 g. 100 ml. of each elutant 1 to 3. Extracts 1 to 4 were made, after development, from successive sections of the column, starting from the bottom.

In the test for tumour-promoting action, Eluates 1 and 2 were pooled, and so were Extracts 1 and 2, and 3 and 4, respectively. They were applied as 0·02 per cent solutions in acetone, with the exception of Eluate 3 which was applied as a 0·01 per cent solution. The results are given in Table IV.

TABLE IV.

| Fraction. | Yields as per cent or resin. | Concentration in acetone applied (%). | Total tumours. | Tumour-bearing mice/survivors. | Tumours per survivor. |
|---|---|---|---|---|---|
| Eluates 1 and 2 | 8·1 | 0·2 | 0 | 0/16 | 0 |
| Eluate 3 | 22·3 | 0·1 | 47 | 11/16 | 3·0 |
| Extracts 1 and 2 | 46·3 | 0·2 | 37 | 9/16 | 2·3 |
| ,, 3 and 4 | 10·1 | 0·2 | 8 | 4/16 | 0·5 |

Sixteen mice per Fraction: Primary treatment: 0·2 ml. 0·15 per cent D.M.B.A./acetone. Interval: 4½ weeks. Secondary treatment: 0·3 ml. of test solution applied to skin weekly for 13 weeks.

Eluate 3 (20 per cent acetone/$CCl_4$) and Extracts 1 and 2 (methanol extracts of the lower half of the column after development) contained the bulk of the active agent or agents. These fractions were therefore chosen for further study.

After some further experiment a method was evolved of which the following is an example.

Fifty-four grams of croton oil in 100 ml. of petroleum ether (B.P. 60–80° C.) was shaken with three successive 25 ml. lots of 90 per cent methanol. The latter were pooled, evaporated in partial vacuum at 60° C., and dried in a vacuum dessicator over anhydrous calcium chloride for 1 day. The yield of resin was 1·8 g.

One and a half grams of this resin was dissolved in $CCl_4$ and deposited on a 60 g. alumina column (Grade II). It was eluted with $CCl_4$, and mixtures of $CCl_4$ and acetone containing successively higher concentrations of acetone.

No appreciable amounts of the material were eluted until the acetone concentration reached 20 per cent. Thereafter successive 50 to 100 ml. lots of 20 per cent acetone/$CCl_4$ eluate were collected separately, and evaporated as described above. These are listed, and their characteristics recorded, in Table V.

TABLE V.

| Fraction. | Elutant. | Volume of elutant (ml.). | Weight of fraction. | Yield as per cent of resin. |
|---|---|---|---|---|
| 1 | 20% acetone/$CCl_4$ | 100 | ·125 | 8·3 |
| 2a | 20% ,, ,, | 50 | ·285 | 19·0 |
| 2b | 20% ,, ,, | 50 | ·191 | 12·7 |
| 3 | 20% ,, ,, | 100 | ·147 | 9·8 |
| 4 | 20% ,, ,, | 100 | ·070 | 4·7 |
| 5 | 20% ,, ,, | 100 | ·044 | 2·9 |
| 6 | 20% ,, ,, | 100 | ·101 | 6·7 |
| 7 | 20% ,, ,, | 100 | ·010 | 0·7 |
| 8 | 50% ,, ,, | 100 | ·006 | 0·4 |
| 9 | 50% ,, ,, | 100 | ·002 | 0·1 |
| Total recovery | | | 1·035 | 68·6 |

Starting material : 1·5 g. resin. Alumina column : 60 g.

Examination of the yield in each fraction shows that the material descends in two main blocks : the first, which is probably complex, comprises Fractions 1 to 5, the second is mainly confined to Fraction 6.

Fraction 1 and Fraction 3, representing the front and rear parts of the first block, and Fraction 6 representing the second block, were separately tested for tumour-promoting action. The results are given in Table VI.

TABLE VI.

| Fraction. | Yield as per cent of resin. | Concentration in acetone applied (%). | Total tumours. | Tumour-bearing mice/survivors. | Tumours per survivor. |
|---|---|---|---|---|---|
| 1 | 8·3 | 0·005 | 98 | 13/16 | 6·1 |
| 3 | 9·8 | ,, | 28 | 7/16 | 1·8 |
| 6 | 6·7 | ,, | 0 | 0/5 | 0·0 |

Sixteen mice per fraction. Primary treatment : 0·2 ml. 0·15 per cent D.M.B.A./acetone. Interval : 3 weeks. Secondary treatment : 0·3 ml. of test solution applied to skin three times per week for 9 weeks.

They shew that the activity is carried by the first block of eluted material, and that the front portion of this is more active than the rear.

Having established the general conditions under which an active substance, or group of substances, could be separated from the resin, an attempt was made to split this material into fractions by further chromatography, using graded elutants.

It was found that no further purification could be effected by the use of acetone/carbon tetrachloride mixtures. Methanol/carbon tetrachloride mixtures appeared more promising. To reduce the amounts of liquids to be handled only small quantities of resin were subjected to chromatography. The work was simplified by the discovery that the material eluted from the column gave a reddish yellow colour, in very dilute solution, in presence of concentrated sulphuric acid. The movement of the bands down the column could be followed by taking samples of eluate at regular intervals, and adding concentrated sulphuric acid.

The following was a typical experiment : ·087 g. of resin, produced as described in Experimental, Section I, was dissolved in about 5 ml. carbon tetrachloride and passed over a 6 g. alumina column made up in carbon tetrachloride.

The column was then eluted, first with $CCl_4$, then with $CCl_4$/methanol mixtures containing rising concentrations of methanol (0 to 1·2 per cent), and finally with 20 per cent acetone in $CCl_4$. Twelve successive fractions were collected. The yields are recorded in Fig. 1.

Fig. 1.

There appears to be three peaks. Frequent application of the $H_2SO_4$ test during elution suggested that a cleaner separation between these peaks had in fact been made than appears from the dry weights recorded. Unfortunately, though this tests reveals very small quantities of these resinous materials, the

Table VII.—*Tests of Methanol/$CCl_4$ Eluates of " Cherbuliez Resin " for Tumour-promoting Activity.*

| Sample. | Yield as per cent of resin. | Concentration in acetone applied (%). | Total tumours. | Tumour-bearing mice/survivors. | Tumours per survivor. |
|---|---|---|---|---|---|
| 1. (Fractions 1 and 2) (see Fig. 1) | 18 | 0·008 | 0 | 0/15 | 0 |
| 2. (Fractions 4 to 7) (see Fig. 1) | 51 | 0·008 | 52 | 8/15 | 3·5 |
| 3. (Fractions 9 to 11) (see Fig. 1) | 17 | 0·008 | 0 | 0/15 | 0 |
| Resin | 100 | 0·02 | 35 | 9/14 | 2·5 |

Fifteen mice in each group. Primary treatment : 0·2 ml. 0·15 per cent D.M.B.A./acetone. Interval : 4 weeks. Secondary treatment : 0·3 ml. of sample in acetone applied to skin 3 times a week for 10 weeks.

depth of colour is too much affected by temperature and time of standing to serve as the basis for a useful quantitative colourimetric assay.

Three samples were formed by pooling Fractions 1 and 2, 4 to 7, and 9 to 11, respectively, and tested for tumour-promoting activity. The results are given in Table VII.

It appears that the tumour-promoting activity is confined to Sample 2. Any activity in Samples 1 and 3 must be of a low order.

IV. *Physical and chemical properties of chromatographic fractions.*

Full descriptions of these substances will be given in a later report. A few general properties will be described here.

All the fractions eluted from alumina columns by methanol/carbon tetrachloride mixtures are resinous materials. Attempts to crystallize them have so far been unsuccessful. It is unlikely that any of them is a pure substance. All are insoluble in water and soluble in organic solvents. They give a reddish colour in dilute solution on addition of concentrated sulphuric acid. They are slowly soluble in 20 per cent aqueous KOH. They take up bromide in $CCl_4$ solution in the cold ; further bromine is taken up on heating, with the liberation of HBr. The iodine number determined by the pyridine dibromide method (Hodgson-Jones and Wheatley, 1952) is variable, but is usually about 50 cg. per g. The active fractions (Eluates 4 to 7, Fig. 1) form white friable foamy deposits on evaporation of the solvent, while the inactive fractions have the appearance of very viscous liquids.

All the samples tested for tumour-promoting activity (Table VII) cause hyperplasia of the epidermis of approximately equal severity at similar concentrations.

### DISCUSSION.

Berenblum (1944, 1954) has speculated on the nature of tumour-promotion by croton oil and other substances. He points out that of many agents which induce hyperplasia, only a few are tumour-promoting, and therefore that the tumour-promoting action of croton oil cannot depend only on its ability to induce hyperplasia.

However no tumour-promoting agent has yet been discovered which does not also induce hyperplasia of the tissue concerned. Iodo-acetic acid and chloroacetophenone, for instance, previously shown in this laboratory to be tumour-promoting in mouse skin (Gwynn and Salaman, 1953), also induce epidermal hyperplasia. In a recent report Daneel and Wissenfels (1955) describe the separation of croton oil, by a chromatographic method, into irritant and non-irritant fractions. They claim that only the non-irritant fraction is tumour-promoting when applied to mouse skin previously treated with methylcholanthrene. The validity of this claim is difficult to assess because of the small number of mice used, and because the criterion for assessment of the irritant property (entzündlichen Wirkung) is not stated. Their work is being repeated in this laboratory.

As judged by microscopical appearance all the croton oil fractions examined in the present work produced hyperplasia of the epidermis, i.e. this property was not confined to the tumour-promoting fractions.

Further work will be needed before the relation between hyperplasia and tumour-promotion is made clear.

The active fraction which has been separated from croton oil is tumour-promoting in mouse skin in far higher dilution than any other substance known. It is therefore important to determine its chemical nature. Efforts to do this have met with considerable difficulties, but are being continued.

### SUMMARY.

The tumour-promoting substance present in croton oil has been concentrated by solvent partition followed by chromatographic analysis. The product obtained, a white amorphous solid, is very much more potent as a tumour-promoter (about fifty times) than the original oil. Some properties of this substance are described.

The author wishes to thank Dr. M. H. Salaman for his helpful criticism and interest, and Dr. D. H. Adams, Dr. G. H. Beaven, and Dr. R. J. C. Harris for many valuable suggestions. He is indebted to D. I. Connell, B. DeBoise, W. J. Milton, and J. Rawlings for skilled technical assistance.

The expenses of this research were partly defrayed out of a block grant from the British Empire Cancer Campaign.

### REFERENCES.

BERENBLUM, I.—(1941) *Cancer Res.*, **1**, 807.—(1944) *Arch. Path.*, **38**, 233.—(1954) *Ibid.*, **14**, 471.

*Idem* AND SHUBIK, P.—(1949) *Brit. J. Cancer*, **3**, 384.

CHERBULIEZ, E., EHNINGER, E. AND BERNHARD, K.—(1932) *Helv. Chim Acta.*, **15**, 658.

DANEEL, R. AND WISSENFELS, N.—(1955) *Naturwissenschaften*, **42**, 128.

GWYNN, R. H. AND SALAMAN, M. H.—(1953) *Brit. J. Cancer*, **7**, 482.

HODGSON-JONES, I. S. AND WHEATLEY, V. R.—(1952) *Biochem. J.*, **52**, 460.

THOMSON, W.—(1930*a*) *J. Hyg., Camb.*, **36**, 24.—(1930*b*) *Ibid.*, **36**, 156.